# Case of Suicide by Opium and Strychnine

**Published:** 1860-11

**Authors:** J. S. Pashley

**Affiliations:** Osceola, Stark Co., Ill.


### ARTICLE 2.

### CASE OF SUICIDE BY OPIUM AND STRYCHNINE.

REPORTED BY J. S. PASHLEY, M. D., OSCEOLA, STARK CO., ILL.

On Sunday, Sept. 2d, 1860, I was called to see A. R. Wells, a native of New York, for some years a resident of Valparaiso, Chili, but then on a visit to friends here. Age 29; stoutly built; in ordinary health for some time previous, though mentally depressed by pecuniary embarrassment, and disabled from a paralytic attack which occurred five years previously, and was repeated three years ago, leaving the left side of the body entirely useless.

From his own statement, made to me after being called, he had, for purposes of self-destruction, taken sixteen pills of his own compounding, containing strychnine gr. 3, opium 1 drachm, and an indefinite quantity of quinine, about 10 o'clock of the evening previous; so that when I saw him at 10 A. M. of the 2d, he had been twenty-four hours under the operation of his dose, resolutely denying to his friends any unusual feelings or sensations, in spite of his strange appearance and actions.

Present appearance, (10 A. M.)—Every evidence of the most extreme cerebral excitement; conjunctiva highly injected; eyes suffused with tears, pupils contracted, and the whole face of a deep red color, mouth and lips dry and clammy, tongue tremulous and covered with a white brownish fur; voice dry, husky, and incoherent in expressions; whole surface hot, with profuse perspiration; body and limbs in violent tremor, and at intervals spasmodic action of all the muscles, alternating with comparative quiet and drowsiness, from which he was easily aroused. Pulse 120, full and forcible; carotid pulsating violently; the respiration labored, and the air of the room redolent of opium. Complained only of feeling "queer."

As speedily as possible administered zinci. sulph. in full emetic doses, in strong coffee, a repetition of which was required twice before full and free emesis could be obtained, by which opium was freely ejected.

Dr. Boardman, of Elmira, was called *en passant.*

11 o'clock.—Patient much more quiet and force of the heart's action somewhat diminished. He now required frequent rousing, but when roused would start violently and remain delirous for some minutes. Gave croton oil gtt. iii, with camph. gr. viij., applied bladder of cold water to head. Perspiration still very profuse.

1 P. M.—Profound stupor suddenly supervened, from which no effort could arouse him. Cold water was applied to the spine and occiput *ad libitum* followed by violent friction shakings, &c., but of no avail.

Muscular system alternately relaxed and rigid; deglutition impossible; pupils contracted still more; breathing stertorous; surface dry and cooler; pulse rapidly sinking; heart's action very irregular.

3 P. M.—Superficial vessels of head and neck suddenly engorged, surface became livid, pulsation at the wrist not readily observable, and those of the heart intermittent. The blood seemed to leave the heart en masse, and rush to the head during the interval of two or three beats, alternating thus for about half an hour, when more regular and rapid action of the heart followed.

4 P. M.—Comparatively quiet, having previously applied sinapism to spine, and blisters to inside of the thighs, an electro-magnetic battery of considerable power was used, with the effect to stimulate muscular and nerve force, and by keeping up the heart's action, maintaining the normal heat of the body to the last moment of life.

Sept. 3d, 8 A. M.—Continued as before, only that on violently shaking, the jaws and eyelids would close, being generally more susceptible to electric force. Respiration sighing; some symptoms of peristaltic action in bowels. Administered enema of croton oil, chlor. soda and lard, with warm water, but which was speedily returned.

1 P. M.—Succeeded in giving croton oil gtt. ij and a small quantity of camphor in water. The same being slowly but effectually swallowed.

2.15 P. M.—While standing by the patient and somewhat cheered by the apparently gradual return of sensibility for the past four or six hours, the pupils suddenly dilated, followed by a slight movement of the body, and death closed the scene.

From the above somewhat lengthy detail, it will be observed that the patient was under the operation of his dose thirteen hours before complete stupor supervened, during which time, as far as observed, he was subjected to a continual struggle for the predominance in effect of the strychnia and opium, the narcotic finally prevailing, so that he remained in the most profound stupor for twenty-five hours and fifteen minutes longer, making in all thirty-eight hours and fifteen minutes before the fatal termination.

A hasty examination of the brain was permitted, nineteen hours after death, in presence of Dr. E. R. Boardman, and J. G. Boardman, medical student.

Found longitudinal sinus and all the superficial vessels unusually engorged, more particularly on the right side. The arachnoid membrane of that side presented a remarkable contrast to its opposite, being highly injected, and consequently very dark in color.

The cavity of the right ventricle was greatly enlarged as compared with its fellow, and its walls much more readily broken down. All the brain matter on the right side was unusually soft, and could with difficulty be handled.

Our conclusions were, that the abnormal condition of the brain was owing to previous disease, only tending to urge on the fatal result of the poisoning.

